# Immunological features of various molecular subtypes of cervical cancer and their prognostic implications in the context of disulfidptosis

**DOI:** 10.3389/fonc.2025.1574911

**Published:** 2025-05-14

**Authors:** Yadan Yao, Xiaomin Yang, Yuanxin Fu, Yinmin Zhang

**Affiliations:** ^1^ Department of Gynecology, Jiaxing University Affiliated Traditional Chinese Medicine (TCM) Hospital, Jiaxing, Zhejiang, China; ^2^ Department of Acupuncture and Massage, Jiaxing University Affiliated Traditional Chinese Medicine (TCM) Hospital, Jiaxing, Zhejiang, China; ^3^ Department of Pediatrics, Jiaxing University Affiliated Traditional Chinese Medicine (TCM) Hospital, Jiaxing, Zhejiang, China

**Keywords:** cervical cancer, disulfidptosis, immune cell infiltration, risk signature, prognostic model

## Abstract

**Objective:**

Cervical cancer ranks among the most prevalent malignancies impacting women globally. Disulfidptosis represents a recently identified pathway of cellular demise, although its role in the context of cervical cancer is not well elucidated. This research investigates the significance of Disulfidptosis-Related Genes (DRGs) within cervical cancer. Furthermore, it aims to analyze the differences in prognosis and immune infiltration among different molecular subtypes.

**Methods:**

We compiled genes associated with cervical cancer and disulfidptosis from a variety of databases to perform a differential expression analysis. Subsequently, the samples are grouped through consensus clustering. To evaluate immune cell infiltration, we employed CIBERSORT. Additionally, immune checkpoint genes (ICGs) were gathered from existing literature and databases, enabling statistical analyses of two subtype samples of cervical cancer (CESC). Following our analyses using GO, KEGG, and GSEA to compare the differences between the two subtypes. Lastly, a prognostic risk model was constructed using LASSO regression and validated using ROC.

**Results:**

This study identified seven key genes: *PCBP3, ARNT, ANP32E, DSTN, CD2AP, EPAS1*, and *ACTN1*.The consensus clustering analysis showed differences in immune cell infiltration and DFS(disease-free survival) among the various clusters. The immune checkpoint gene *CXCL1* displayed highly significant statistical differences between subtype A (Cluster 1) and subtype B (Cluster 2) in cervical cancer (CESC) samples. The gene set enrichment analysis identified the negative regulation of peptidase activity and the IL-17 signaling pathway, which link to subtype-specific differentially expressed genes (DEGs).

**Conclusion:**

Statistical analysis of the various subtypes of CESC samples highlighted the importance of subtype-specific therapeutic targets. Additionally, it seeks to enhance the accuracy of prognostic predictions, thereby establishing a foundation for the formulation of personalized treatment approaches.

## 1 Introduction

In 2022, there were over 662,000 new cases reported globally, resulting in around 349,000 deaths (1). Annually, cervical cancer is responsible for nearly 350,000 fatalities among women (2). Although various strategies such as vaccination, screening, and treatment have been introduced, many challenges remain that impede the effective implementation of these interventions (3, 4). It is critical to urgently explore the characteristics of cervical cancer patients and to identify essential prognostic markers and potential therapeutic targets (5–7). Therefore, it is essential to explore optimal treatment approaches by studying the molecular characteristics and prognostic models of cervical cancer subtypes.

Increasing attention is being given to the mechanisms of cell death in contemporary cancer research (8). Notably, the significance of *SLC7A11* in disulfidptosis has gained substantial attention (9). Researchers are exploring the cell death mechanism associated with the increased expression of *SLC7A11* under glucose deprivation conditions. This specific form of cell death is primarily associated with an imbalance in the intracellular NADPH/NADP+ ratio and the excessive accumulation of disulfides. When NADPH levels are diminished, the conversion of cysteine within the cells becomes ineffective, resulting in heightened disulfide stress that ultimately initiates cell death. Disulfidptosis is a newly discovered mechanism of cell death. Identifying Disulfidptosis-Related Genes mechanisms of cell death provides new insights for cancer therapy, especially in targeting tumors that exhibit elevated expression of *SLC7A11* (10, 11). This insight introduces new targeted therapeutic strategies for cancer treatment, revealing potential avenues for intervention. However, the influence of disulfidptosis on cervical cancer remains poorly understood, highlighting the imperative for further investigation in this domain. While prior studies have pinpointed various genes linked to cervical cancer (12, 13), the characteristics of these genes across different subtypes have not been exhaustively examined or comprehended. This study is primarily centered on cervical cancer and genes associated with disulfidptosis. Through the exploration of these associations, our objective is to deepen our comprehension of the molecular pathways involved in cervical cancer. We are conducting research to identify prognostic markers that can assist in diagnosing cervical cancer and assessing their clinical significance. By studying the relationship between cervical cancer and disulfidptosis-related genes, we aim to identify precise treatment targets. We analyzed disulfidptosis-related genes in cervical cancer subtypes using the R package limma for differential expression and consistency clustering to delineate the subtypes.

This methodological approach is advantageous as it systematically reveals gene expression patterns and their correlations with clinical features, thereby enriching our understanding of the underlying biological mechanisms. Moreover, we seek to examine the biological distinctions among the different molecular subtypes. By integrating these methodologies, our goal is to refine the classification of patients based on their molecular attributes. Such refinement may foster the creation of more personalized therapeutic strategies, potentially leading to improved clinical outcomes for individuals diagnosed with cervical cancer.

## 2 Methods

### 2.1 Data acquisition

The cervical cancer (CESC) dataset was procured from TCGA (the cancer genome atlas) database using the “TCGAbiolinks” package (14). This dataset served as our testing set for analysis. This dataset served as our primary analysis set, encompassing 245 cervical cancer samples along with clinical data, in addition to three control samples presented in Counts format. The dataset was standardized to the FPKM format. Clinical information was sourced from the UCSC Xena database (15), with specific details outlined in **Table 1**. Due to the restricted number of control samples available within the TCGA cervical cancer dataset, we supplemented our analysis with normal tissue samples extracted from the Genotype-Tissue Expression (GTEx) database (16). After procuring ten control samples in counts-format sequencing data, we integrated the FPKM-format sequencing data from both TCGA and GTEx, thereby establishing a comprehensive cervical cancer dataset designated as GTEX-TCGA-CESC. The final analysis incorporated 245 cervical cancer samples paired with clinical information alongside 13 control samples, yielding a robust test set.

**Table 1:** Baseline table with CESC patients characteristics.

Characteristics	Overall
Age, median (IQR)	46 (37, 55)
Stage, n (%)	
Stage I	142 (58%)
Stage III	34 (13.9%)
Stage II	56 (22.9%)
Stage IV	13 (5.3%)

CESC, Cervical Cancer.

We used the “GEOquery” R package (17) to retrieve the cervical cancer dataset GSE44001 (18) from the gene expression omnibus database(GEO) (19). As detailed in **Table 2**, the GSE44001 dataset includes samples of human cervical tissue and is associated with the GPL14951 platform, which comprises 262 non-recurrent and 38 recurrent cervical cancer samples included in our study.

**Table 2:** GEO microarray chip information.

	GSE44001
Platform	GPL14951
Species	Homo sapiens
Tissue	Cervical
Samples in CESC group	262
Samples in Control group	38
Reference	PMID: 24145113

GEO, Gene Expression Omnibus; CESC, Cervical Cancer.

Additionally, we accessed the genecards database (20), from which we obtained a list of genes associated with disulfidptosis, referred to as Disulfidptosis-Related Genes (DRGs).By utilizing “Disulfidptosis” as the search keyword, we focused on “Protein Coding” genes, identifying a total of eight DRGs. Furthermore, we conducted a literature search for “Disulfidptosis” on the PubMed website (21), which yielded 106 DRGs. By integrating the data, we identified 108 DRGs detailed in **Supplementary Table S1**, and we normalized the GSE44001 dataset using the “limma” R package (22).

### 2.2 Differentially expressed genes related to cervical cancer

The GTEx-TCGA-CESC dataset is divided into the CESC group and the Control group. Thresholds were set (p < 0.05,|logFC| > 0.58) to identify DEGs, the “limma” and “ggplot2” packages were used here.

### 2.3 Identification of prognostic genes related to cervical cancer via univariate Cox survival analysis

To construct a prognostic risk model for the cervical cancer dataset, we first performed Cox regression analysis to evaluate the impact of genes on prognosis.

Next, we identified independent prognostic factors, which we referred to as Cox genes. We screened for significantly expressed genes in GSE44001 using differential analysis (p < 0.05, |logFC| > 0.58), referred to as differentially expressed genes (DEGs). Subsequently, we compared the DEGs with genes related to disulfide bond death and Cox prognostic genes. We used a Venn diagram to display their intersection to highlight key genes. Throughout the analysis, we utilized the “survival” and “pheatmap” packages in R.

### 2.4 Development of TCGA cervical cancer subtypes

We identified different disease subtypes in CESC samples from the GTEx-TCGA-CESC dataset by applying consensus clustering with the R package ConsensusClusterPlus (23), emphasizing key genes. During this procedure, the cluster count was established within a range of 2 to 9. A total of 80% of the overall samples underwent resampling 50 times, employing the parameters clusterAlg = “pam” and distance = “euclidean”. Following this, the variations in expression levels of crucial genes across distinct disease subtypes were examined utilizing expression value heat maps alongside group comparison plots.

### 2.5 Kaplan-Meier survival analysis and correlation analysis of distinct molecular subtypes in cervical cancer

First, we developed a clinical feature distribution map that shows the clinical characteristics of different molecular subtypes and the expression of intersecting genes. To assess the difference in disease-free survival (DFS) between samples of cervical squamous cell carcinoma (CESC) subtype A (cluster 1) and subtype B (Cluster 2) at different time intervals in the cervical cancer dataset, we performed Kaplan-Meier (KM) curve (24) analysis using the “survival” package in R. Additionally, the Spearman correlation coefficient was used to evaluate expression levels in CESC samples derived from the GTEx-TCGA-CESC dataset. Conclusions were illustrated in a correlation heatmap.

### 2.6 Analysis of immune infiltration

CIBERSORT (25) utilizes linear support vector regression to analyze transcriptomic expression matrices, allowing for the estimation of immune cell composition and abundance in mixed samples. We applied the CIBERSORT algorithm to merge the immune cell characteristic gene matrix and filtered the results to keep only immune cells with enrichment scores above zero. This process resulted in an immune cell infiltration matrix for samples in the cervical cancer dataset. We used the Spearman method to calculate the correlation between immune cells and examined their relationships with key genes. To improve result visualization, we utilized the “ggplot2” and “pheatmap” packages.

### 2.7 Analysis of immune checkpoints, IPS, and TIDE

Immune checkpoint genes (ICGs) represent important ligand-receptor pairs that significantly regulate immune responses, help maintain immune homeostasis, and prevent autoimmunity. Cancer therapy has seen significant success with treatments that target these genes. This is particularly true for solid tumors. We identified six immune checkpoint genes (ICGs)—*CXCL1, CXCL10, CXCL11, CCL8, CCL13*, and *CCL18 (* 26)—and 28 immunogenic cell death (ICD) genes (27) from the published literature, the specific gene names are listed in **Supplementary Table S2**. We employed the Mann-Whitney U test to analyze the expression levels of ICGs and ICD genes across various CESC subtypes in the cervical cancer dataset (GTEx-TCGA-CESC).

### 2.8 GSEA

Perform differential analysis on the cervical cancer dataset (GTEx-TCGA-CESC) using “Limma” to compare subtype A (Cluster 1) with subtype B (Cluster 2). Then, create a volcano plot using “ggplot2” to illustrate the differentially expressed genes (SSDEGs) with |log2FC| > 0.58 and adj.p-value < 0.05. Next, use “pheatmap” to generate a heatmap of the expression of these genes. Finally, conduct gene set enrichment analysis (GSEA) using “clusterProfiler” R package (28). The analysis parameters include a seed of 2020, gene set size limits between 10 and 500. Gene set enrichment analysis (GSEA) was performed using the gene set c2.all.v2022.1.Hs.symbols.gmt obtained from the Molecular Signatures Database (MSigDB) (29). The screening criteria for GSEA were adj.p < 0.05. The p-value correction method used was Benjamini-Hochberg (BH).

### 2.9 Gene ontology and Kyoto encyclopedia of genes and genomes pathway enrichment analysis

Gene Ontology (GO) analysis (30) is a widely used method for functional enrichment studies that include Biological Process (BP), Cellular Component (CC), and Molecular Function (MF). Researchers widely use the Kyoto Encyclopedia of Genes and Genomes (KEGG) (31) database to store information about genomes, biological pathways, diseases, and drugs. We employed the R package “clusterProfiler” to conduct GO and KEGG enrichment analyses on subtype-specific SSDEGs. We set entry screening criteria of adjusted p-values (adj.p) < 0.05 and a false discovery rate (FDR) value (q value) < 0.25, using the Benjamini-Hochberg (BH) correction method.

### 2.10 Construction of prognostic risk model for cervical cancer

We began by conducting univariate Cox regression analysis on the cervical cancer dataset (GTEx-TCGA-CESC) to identify variables with a p-value less than 0.05 for the subsequent multivariate Cox regression analysis. We performed LASSO regression analysis on the subtype differential genes (SSDEGs) identified in the univariate Cox regression model, using family = “cox” as a parameter to select genes for the prognostic risk model. In our analysis, we utilized the R packages “survival” and “glmnet”. Finally, we calculated the risk score (RiskScore) using the following formula based on the risk coefficients obtained from the LASSO regression analysis:


$$\text{Risk}score\;=\;\sum_{i}Coefficient\;\left(gene_{i}\right)*mRNA\;Expression\;\left(gene_{i}\right)$$

### 2.11 Prognostic analysis of cervical cancer prognostic risk model

The dataset categorizes cervical cancer samples into high-risk (HighRisk) and low-risk (LowRisk) groups according to the median risk score. We plot time-dependent ROC curves based on the LASSO risk score (RiskScore) and disease-free survival (DFS). The area under the curve (AUC) is then calculated to predict the 1, 2, and 3-year survival outcomes for the cervical cancer (CESC) group. We perform Kaplan-Meier (KM) curve analysis to assess differences in disease-free survival (DFS).We visualize the results of univariate and multivariate Cox regression analyses with a forest plot, followed by presenting the multivariate Cox regression results in a nomogram to illustrate the relationship between risk score (RiskScore) and clinical information. The results of the multivariate Cox regression analysis are presented in a nomogram to show the relationship between the risk score (RiskScore) and clinical information included in the multivariate Cox regression model. We visualize the results of univariate and multivariate Cox regression analyses with a forest plot, followed by presenting the multivariate Cox regression results in a nomogram to illustrate the relationship between risk score (RiskScore) and clinical information. Through calibration analysis, we draw the calibration curve to evaluate the predictive accuracy and discrimination of the prognostic risk model using the LASSO risk score (RiskScore). We utilize the risk score (RiskScore) to create a Decision Curve Analysis (DCA) plot to assess the accuracy and discrimination of the prognostic risk model for cervical cancer (CESC). In this study, we used the packages “survivalROC,” “survival,” “rms,” and “ggDCA.”

### 2.12 Model validation of GSE44001 in dataset

We validated our model using the GSE44001 dataset and calculated the LASSO risk score (RiskScore) from the model’s coefficients. This score was crucial for assessing the prognostic risk model’s accuracy in cervical squamous cell carcinoma (CESC). Next, plot the time-dependent ROC curves using the LASSO risk score (RiskScore) and DFS to predict the survival outcomes, defined as the likelihood of survival without disease recurrence, for CESC groups at 1, 2, and 3 years.

### 2.13 Gene set variation analysis

To assess the accuracy of the prognostic risk model for cervical cancer (CESC), we first calculated the risk score (RiskScore) using gene expression and LASSO coefficients derived from the GSE44001 dataset. We utilized the R package “survivalROC” to create time-dependent ROC curves, which illustrate the diagnostic performance of our prognostic risk model over time. We calculated the area under the ROC curve (AUC) to predict the survival rates of the cervical cancer (CESC) group at 1, 2, and 3 years.

### 2.14 Statistical analysis

The independent Student’s T-Test and Mann-Whitney U test are used to compare two groups, while the Kruskal-Wallis test is used for comparisons involving three or more groups. The results are calculated using Spearman correlation analysis to determine the correlation coefficients between different molecules, with a p value < 0.05 considered statistically significant.

## 3 Results

### 3.1 Technology roadmap

**Figure 1:**
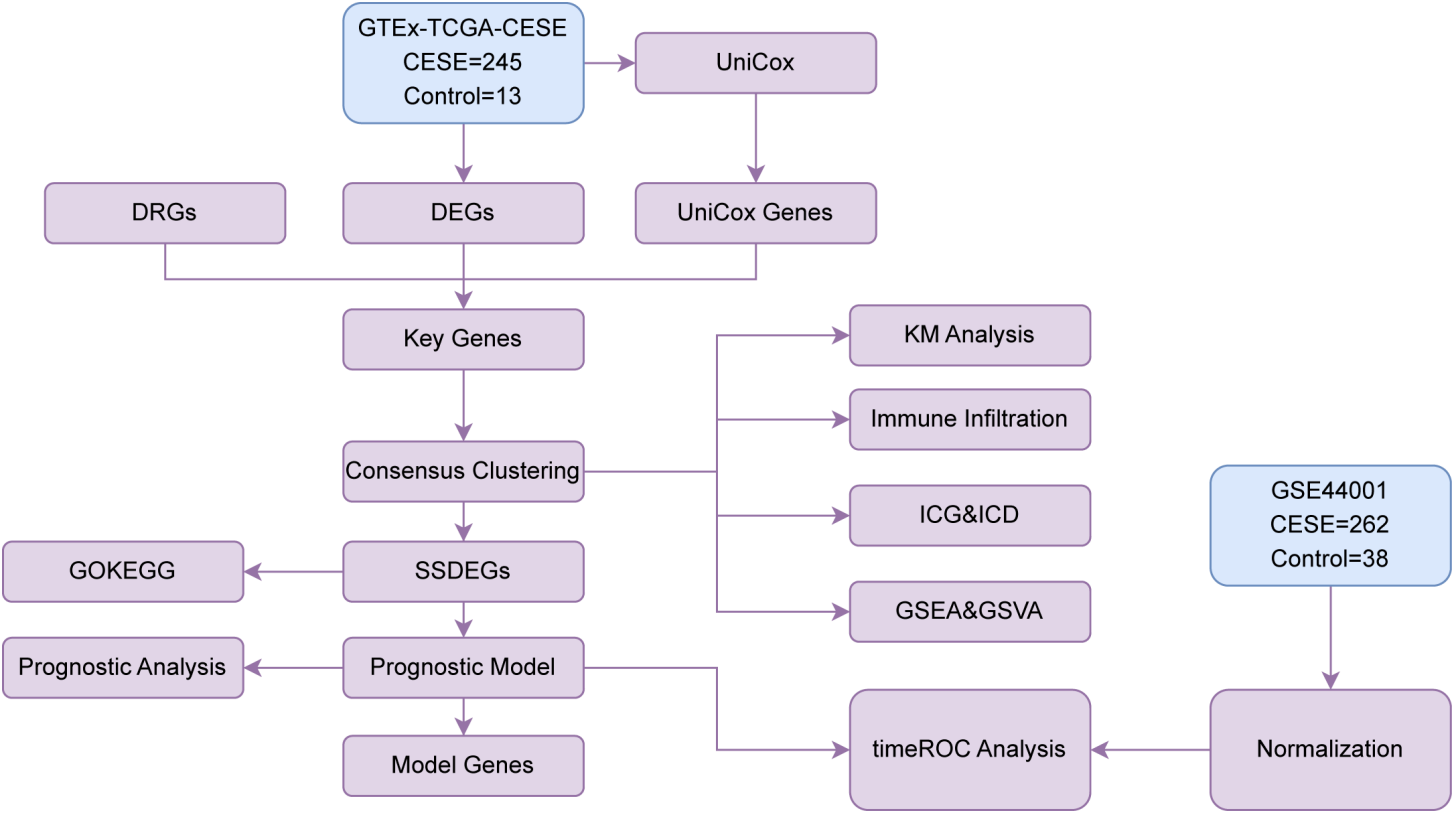
Flow chart for the comprehensive analysis. TCGA, The Cancer Genome Atlas; CESC, Cervical Cancer; DEGs, Differentially Expressed Genes; DRGs, Disulfidptosis-Related Genes; Unicox, Univariate Cox; GSEA, Gene Set Enrichment Analysis; ROC, Receiver Operating Characteristic; GO, Gene Ontology; KEGG, Kyoto Encyclopedia of Genes and Genomes; SSDEGs, Subtype-specific differentially expressed genes; ICG, Immune Checkpoint Genes; ICD, Immunogenic Cell Death; GSVA, Gene Set Variation Analysis.

Flow Chart for the Comprehensive Analysis as shown in **Figure 1**.

### 3.2 Differentially expressed genes related to cervical cancer

**Figure 2:**
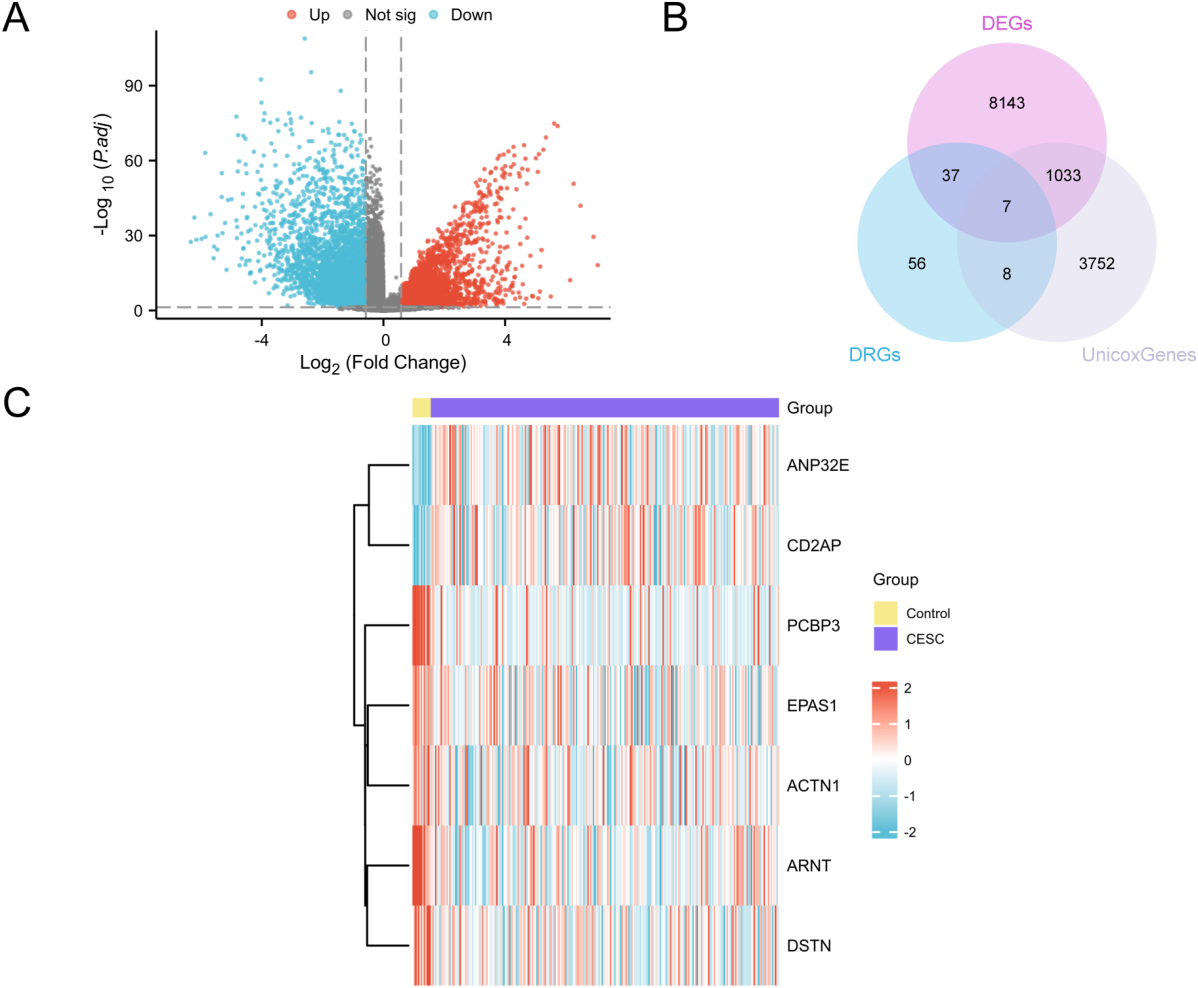
Differential gene expression analysis. **(A)** Volcano plot of differentially expressed genes analysis between the CESC group and the Control (Control) group in the GTEx-TCGA-CESC. **(B)** DEGs, DRGs and univariate Cox gene intersection Venn diagram in the GTEx-TCGA-CESC. **(C)** Heat map of Key Genes in the GTEx-TCGA-CESC. The CESC group is purple, and the Control (Control) group is yellow. In the heat map, red represents high expression and blue represents low expression.

The GTEx-TCGA-CESC dataset was separated into cervical cancer and control groups for differential analysis. We identified 9,220 DEGs that met the criteria of |logFC| > 0.58 and adjusted p-value < 0.05. Among them, 4,004 genes were upregulated and 5,176 genes were downregulated, as shown in a volcano plot (**Figure 2A**). To construct a prognostic risk model for CESC, we performed univariate Cox regression analysis using clinical information from the CESC group, analyzing a total of 4,800 genes (P less than 0.05). Detailed information is provided in **Supplementary Table S3**. We identified key genes by analyzing the intersection of DEGs, DRGs, and genes from the univariate Cox analysis that met the specified threshold. A Venn diagram was created for visualization (**Figure 2B**), identifying seven key genes: *PCBP3, ARNT, ANP32E, DSTN, CD2AP, EPAS1*, and *ACTN1*.Finally, we analyzed the expression levels of key genes across different sample groups and created a heatmap using the R package pheatmap (**Figure 2C**).

### 3.3 Construction of TCGA Cervical Cancer Subtypes

**Figure 3:**
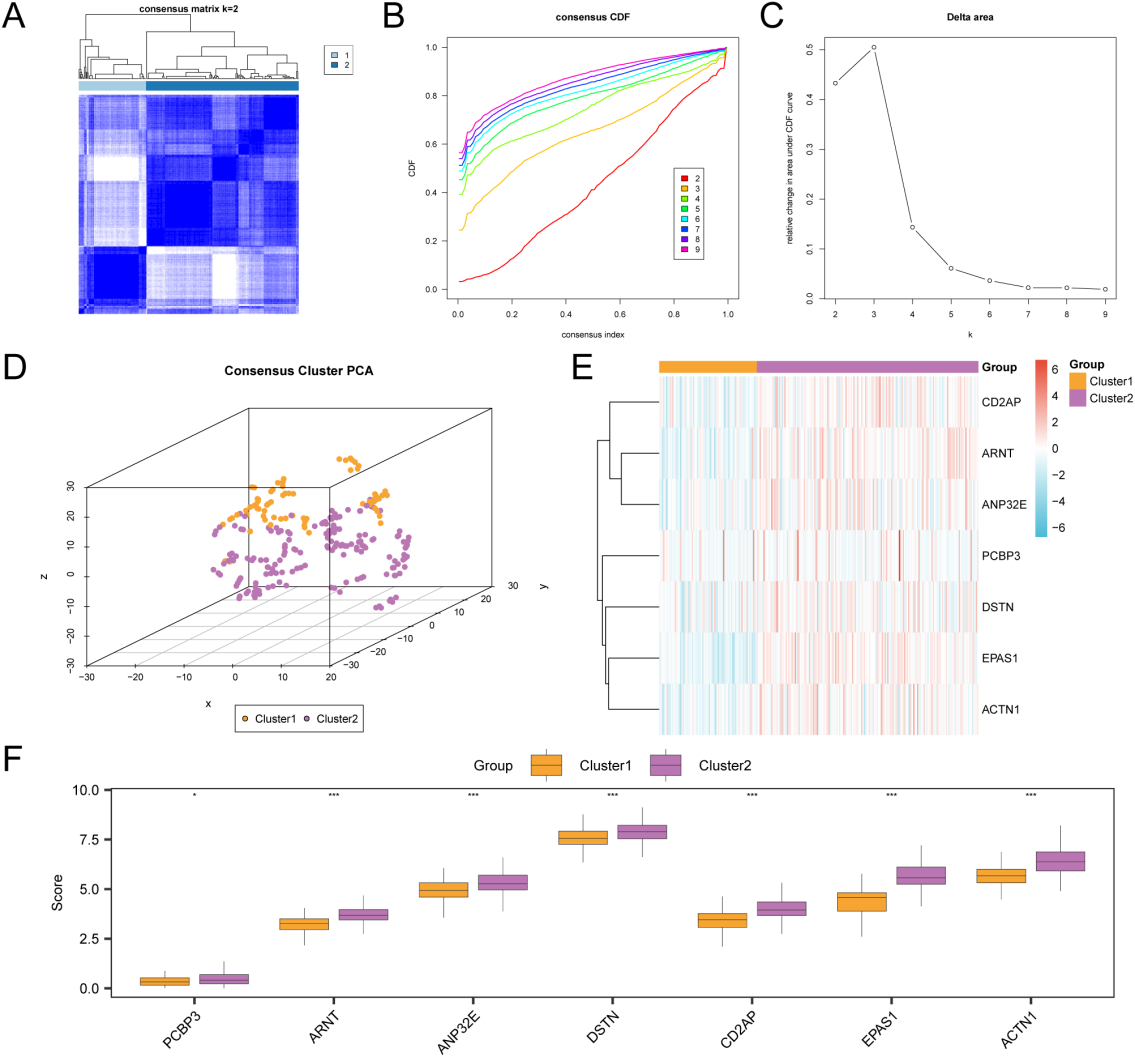
Consensus clustering analysis for CESC. **(A)** Consensus clustering results of CESC samples in the GTEx-TCGA-CESC. B-C. Consistency cumulative distribution function (CDF) plot **(B)** and Delta plot **(C)** of consistency clustering analysis. **(D)** 3D t-SNE cluster map of two disease subtypes of cervical cancer (CESC). **(E)** Heat map of expression values of Key Genes in CESC subtypes. **(F)** Group comparison map of Key Genes between the two subtypes of CESC. PCA, Principal Component Analysis. * represents p value < 0.05, statistically significant; *** represents p value < 0.001, highly statistically significant. Orange is subtype A (Cluster1) and purple is subtype B (Cluster2).

This study examined disease subtypes in the cervical cancer dataset (GTEx-TCGA-CESC) by analyzing the expression levels of seven key genes. Through consistent clustering analysis, we identified two subtypes: subtype A (Cluster 1, 75 samples) and subtype B (Cluster 2, 170 samples) (**Figures 3A–C**). Additionally, the 3D t-SNE clustering diagram demonstrates significant differences between the two subtypes (**Figure 3D**). Next, use the R package “pheatmap” to draw a heatmap showing the expression differences of key genes in the two subtypes of CESC (**Figure 3E**). Finally, to further validate the expression differences of key genes in the CESC disease subtypes, a grouped comparison chart (**Figure 3F**) is presented to display the expression levels of key genes, illustrating the differential analysis results between the two subtypes of CESC. The comparison chart shows that the expression of *PCBP3* is statistically significant (p < 0.05), while the expressions of *ACTN1, ANP32E, ARNT, CD2AP, DSTN*, and *EPAS1* are highly significant (p < 0.001).

### 3.4 KM survival analysis and correlation analysis of different molecular subtypes in cervical cancer

**Figure 4:**
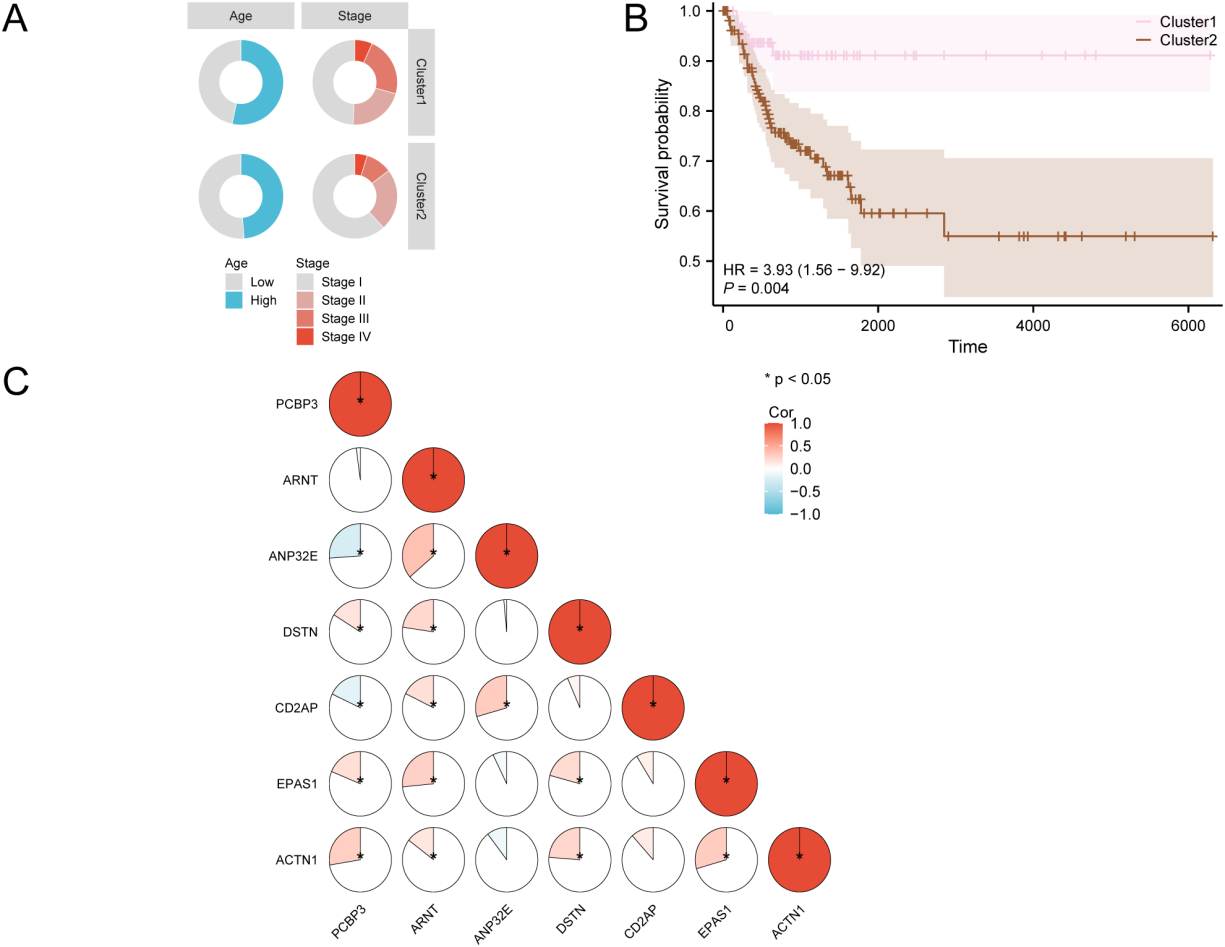
KM survival analysis of CESC. **(A)** Distribution of clinical features in different subtypes. **(B)** Prognostic KM curves for disease-free survival (DFS) of CESC samples based on subtype grouping in the GTEx-TCGA-CESC. **(C)** Correlation heatmap of Key Genes in the GTEx-TCGA-CESC. KM, Kaplan-Meier method.

We plotted the distribution of clinical characteristics for different subtypes (**Figure 4A**). This plot illustrates the expression of intersecting genes, which aids in understanding the biological features of these subtypes. We conducted a prognostic Kaplan-Meier curve analysis based on disease-free survival and subtype grouping from the cervical cancer dataset (**Figure 4B**). The results indicate that there is a statistically significant difference in DFS between the two subtypes (p < 0.01). We assessed the correlations of seven key genes in the cervical cancer dataset and created a heatmap (**Figure 4C**), which reveals that these genes primarily show positive correlations.

### 3.5 Immune infiltration analysis of cervical cancer subtypes

**Figure 5:**
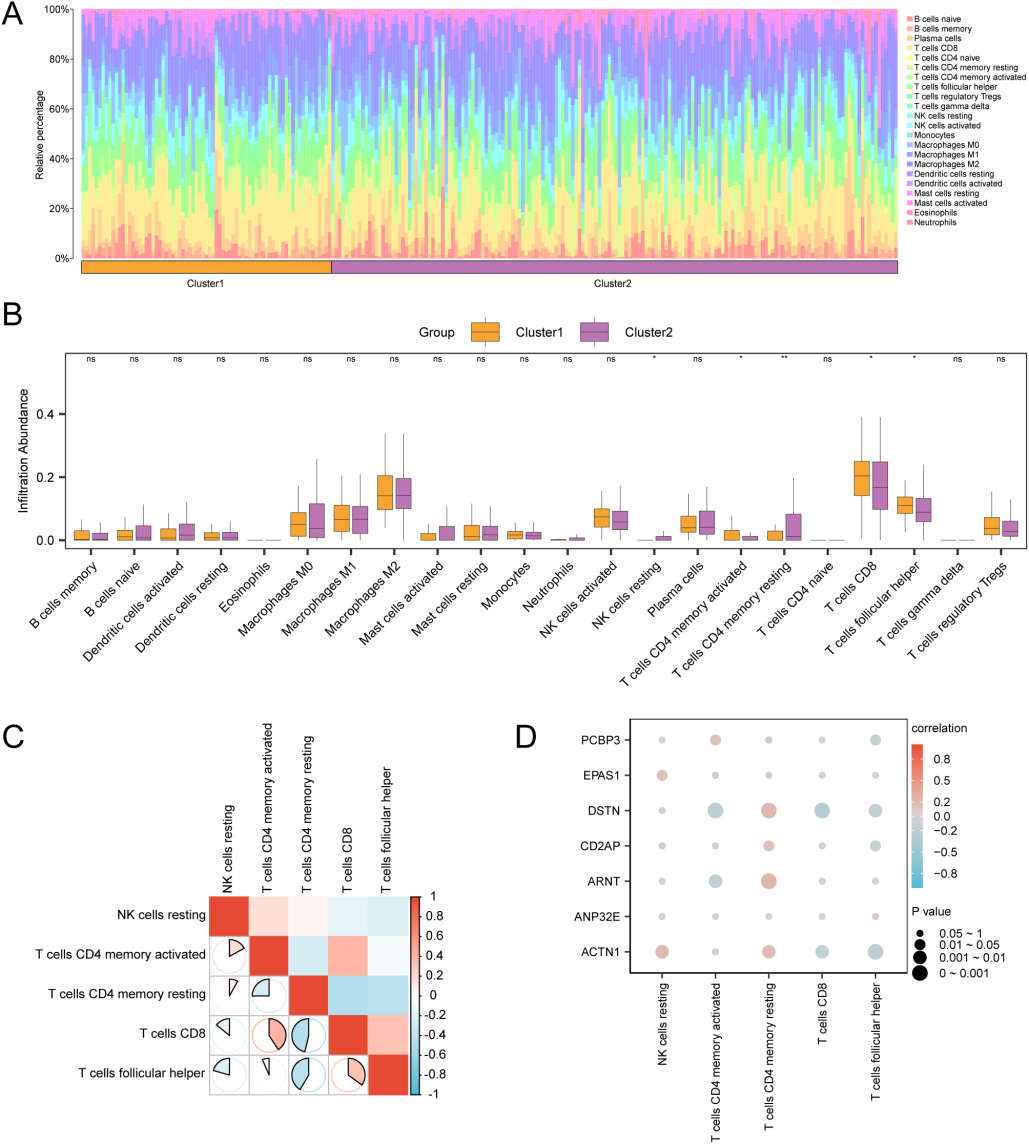
Immune infiltration analysis by CIBERSORT algorithm. **(A, B)** The proportion of immune cells in CESC samples in the GTEx-TCGA-CESC bar graph **(A)** and group comparison graph **(B)**. **(C)** Correlation heat map of immune cells in CESC samples in the GTEx-TCGA-CESC. **(D)** Bubble plot of correlation between immune cell infiltration abundance and Key Genes in CESC samples in GTEx-TCGA-CESC dataset. ns stands for p value ≥ 0.05, not statistically significant; * represents p value < 0.05, statistically significant; ** represents p value < 0.01 and highly statistically significant. The absolute value of correlation coefficient (r value) below 0.3 was weak or no correlation, between 0.3 and 0.5 was weak correlation, between 0.5 and 0.8 was moderate correlation, and above 0.8 was strong correlation. Orange is subgroup A (Cluster1), purple is subgroup B (Cluster2). Positive correlations are shown in red and negative ones in blue. The depth of the color represents the strength of the correlation.

We used the CIBERSORT algorithm to calculate the abundance of 22 types of immune cell infiltration in the GTEx-TCGA-CESC cervical cancer dataset. A bar chart (**Figure 5A**) displayed the proportions of immune cells, while a comparison chart (**Figure 5B**) illustrated the differences in immune cell infiltration abundance between groups. The results showed that five types of immune cells were statistically significant (p < 0.05): *CD8 T* cells, resting memory *CD4 T* cells, activated memory *CD4 T* cells, follicular helper T cells, and resting NK cells. Following this, we illustrated the correlation of infiltration abundance among these five immune cell types using a correlation heatmap (**Figure 5C**). Most immune cells exhibited strong correlations, particularly *CD8 T* cells, which had a correlation coefficient (r) of -0.463 with resting memory *CD4 T* cells (p < 0.05). Finally, we presented a correlation bubble chart (**Figure 5D**) to illustrate the relationship between key genes and immune cell infiltration abundance. Notably, the gene *DSTN* had an r = -0.285 with *CD8 T* cells, p < 0.05.

### 3.6 ICG&ICD analysis

**Figure 6:**
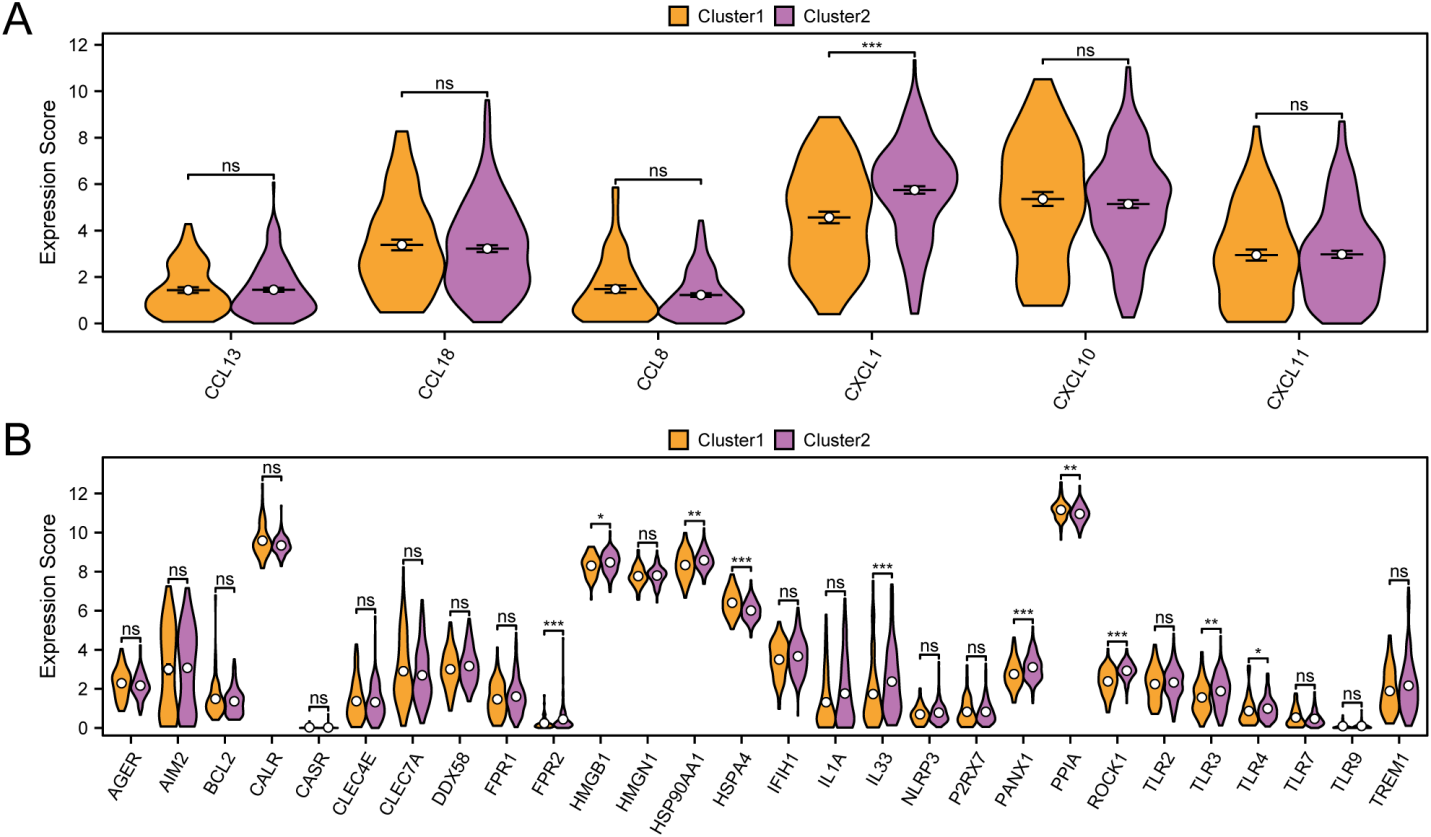
Immune checkpoint genes, immunogenic cell death genes analysis. **(A)** Group comparison of ICG between subtype A (Cluster1) and subtype B (Cluster2) of CESC samples from the GTEx-TCGA-CESC. **(B)** Group comparison of ICD genes between subtype A (Cluster1) and subtype B (Cluster2) of CESC samples from the GTEx-TCGA-CESC. ns represents p value ≥ 0.05, not statistically significant; * represents p value < 0.05, statistically significant; ** represents p value < 0.01, highly statistically significant; *** represents p value < 0.001 and extremely statistically significant. Orange is subtype A (Cluster1) and purple is subtype B (Cluster2).

For the immune checkpoint genes (ICG) and immunogenic cell death genes (ICD) analysis, we compiled relevant genes from existing literature and the GeneCards database. By cross-referencing these with all genes in the GTEx-TCGA-CESC dataset, we constructed a matrix that included six ICG—specifically *CXCL1, CXCL10, CXCL11, CCL8, CCL13*, and *CCL18*—alongside their respective expression levels. The immune checkpoint gene CXCL1 shows statistically significant differences between subtype A (Cluster 1) and subtype B (Cluster 2) in cervical cancer (CESC) samples (p value < 0.001) (**Figure 6A**).

We evaluated the statistical differences in immunogenic cell death (ICD) genes between subtype A (Cluster 1) and subtype B (Cluster 2) (**Figure 6B**). The results revealed that several ICD genes, including *FPR2, HSPA4, IL33, PANX1*, and *ROCK1*, demonstrated notable differences between the two subtypes, each with p-values less than 0.001. Furthermore, *HMGB1* and *TLR4* revealed significant differences between subtype A (Cluster 1) and subtype B (Cluster 2), with p-values less than 0.05.

### 3.7 GSEA for subtype grouping

**Figure 7:**
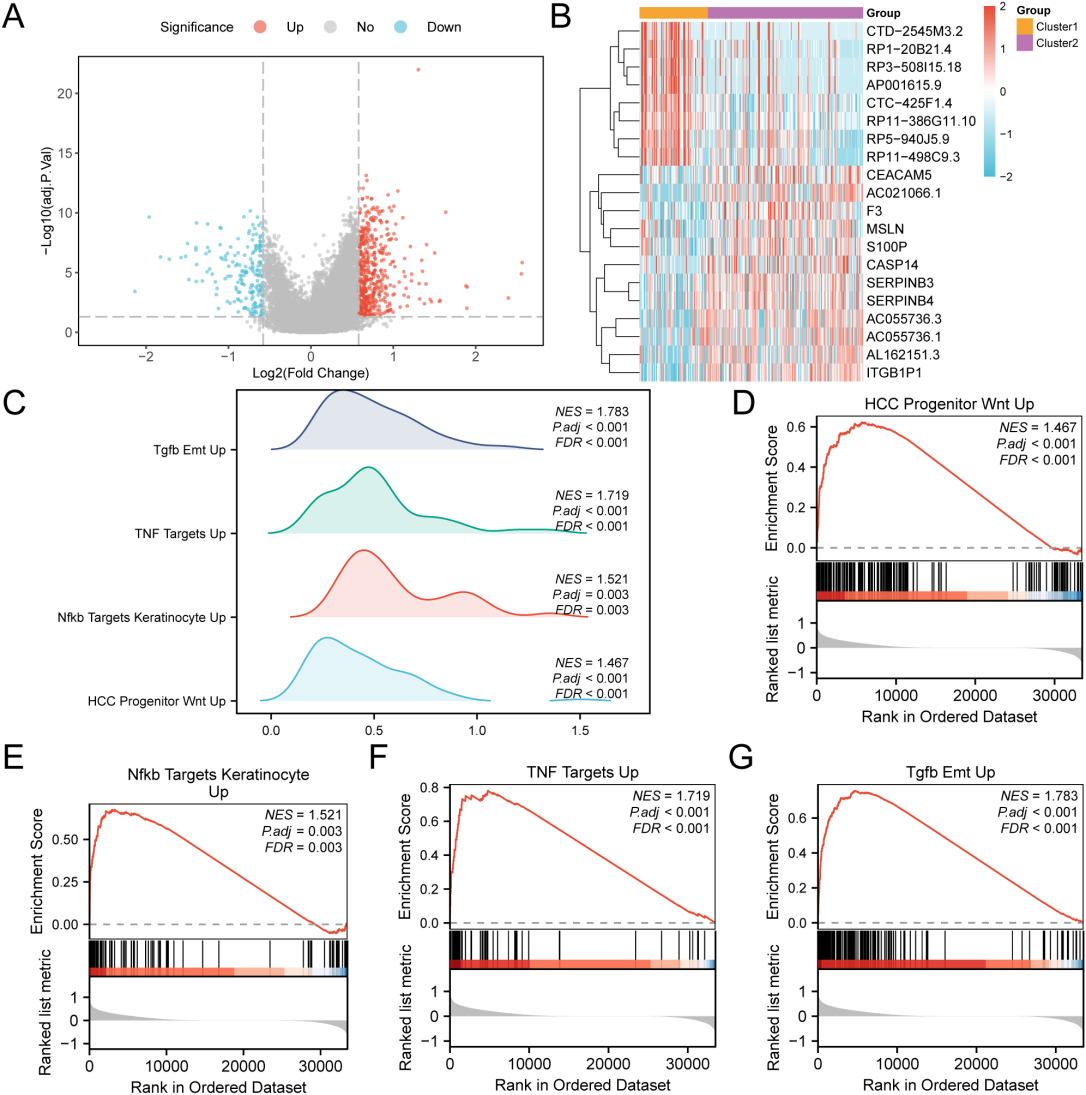
GSEA for risk group. **(A)** Volcano plot of differentially expressed genes analysis of subtype A (Cluster1) and subtype B (Cluster2) in CESC samples in the GTEx-TCGA-CESC. **(B)** Heat map of expression values of differentially expressed genes of subtype A (Cluster1) and subtype B (Cluster2) of cervical cancer (CESC) samples. **(C)** Four biological functions mountain map display of GSEA of GTEx-TCGA-CESC. D-G. GSEA showed that genes in the GTEx-TCGA-CESC were significantly enriched in HCC Progenitor Wnt Up **(D)**, Nfkb Targets Keratinocyte Up **(E)**, and NFKB targets keratinocyte UP **(E)**. TNF Targets Up **(F)**, Tgfb Emt Up **(G)**. In the heat map, orange represents subtype A (Cluster1) and purple represents subtype B (Cluster2). In the heat map, red represents high expression and blue represents low expression. The redder the color, the smaller the adj.p value, and the bluer the larger the adj.p value. The screening criteria of GSEA were adj.p < 0.05 and FDR value (q value) < 0.25, and the p value correction method was Benjamini-Hochberg (BH).

The analysis of the cervical cancer dataset (GTEx-TCGA-CESC) found 677 genes that are differentially expressed (p < 0.05 and |logFC| > 0.58). Among them, 489 were upregulated and 188 were downregulated. We created a volcano plot (**Figure 7A**) and a heatmap (**Figure 7B**) to visualize the data. Gene set enrichment analysis (GSEA) revealed the relationships of all genes with biological processes, cellular components, and molecular functions (**Figure 7C**). The specific results are shown in **Table 3**. The analysis also indicated significant enrichment in specific signaling pathways, highlighting the upregulation of HCC precursor Wnt (**Figure 7D**), Nfkb target keratinocytes (**Figure 7E**), TNF targets (**Figure 7F**), and Tgfb Emt (**Figure 7G**).

**Table 3:** Results of GSEA for GTEx-TCGA-CESC.

ID	setSize	enrichmentScore	NES	pvalue	p.adjust	qvalue
DACOSTA_UV_RESPONSE_VIA_ERCC3_COMMON_DN	451	0.76	1.81	1.00E-10	8.65E-09	6.65E-09
ZWANG_CLASS_3_TRANSIENTLY_INDUCED_BY_EGF	223	0.76	1.80	1.00E-10	8.65E-09	6.65E-09
FOROUTAN_TGFB_EMT_UP	190	0.76	1.78	1.00E-10	8.65E-09	6.65E-09
FOROUTAN_PRODRANK_TGFB_EMT_UP	183	0.75	1.77	1.00E-10	8.65E-09	6.65E-09
BILD_CTNNB1_ONCOGENIC_SIGNATURE	77	0.79	1.76	2.20E-09	1.41E-07	1.08E-07
HUANG_DASATINIB_SENSITIVITY_UP	77	0.79	1.76	2.57E-09	1.60E-07	1.23E-07
GABRIELY_MIR21_TARGETS	278	0.74	1.76	1.00E-10	8.65E-09	6.65E-09
LIN_SILENCED_BY_TUMOR_MICROENVIRONMENT	101	0.77	1.76	2.80E-10	2.21E-08	1.70E-08
ANASTASSIOU_MULTICANCER_INVASIVENESS_SIGNATURE	64	0.80	1.76	7.30E-08	3.43E-06	2.64E-06
DAZARD_RESPONSE_TO_UV_NHEK_DN	290	0.74	1.76	1.00E-10	8.65E-09	6.65E-09
WP_MIRNA_TARGETS_IN_ECM_AND_MEMBRANE_RECEPTORS	42	0.84	1.75	9.37E-07	3.15E-05	2.42E-05
REACTOME_NON_INTEGRIN_MEMBRANE_ECM_INTERACTIONS	59	0.80	1.75	1.10E-07	4.96E-06	3.81E-06
AMIT_SERUM_RESPONSE_60_MCF10A	56	0.80	1.74	4.52E-07	1.73E-05	1.33E-05
GENTILE_UV_RESPONSE_CLUSTER_D4	53	0.81	1.74	4.80E-07	1.79E-05	1.38E-05
DE_YY1_TARGETS_DN	91	0.77	1.73	6.82E-09	3.95E-07	3.04E-07
SIMBULAN_UV_RESPONSE_IMMORTALIZED_DN	31	0.87	1.73	3.05E-06	8.71E-05	6.70E-05
RODRIGUES_THYROID_CARCINOMA_DN	75	0.77	1.73	3.44E-08	1.70E-06	1.31E-06
PHONG_TNF_TARGETS_UP	63	0.78	1.72	4.66E-07	1.75E-05	1.35E-05
HINATA_NFKB_TARGETS_KERATINOCYTE_UP	84	0.67	1.52	2.26E-04	3.32E-03	2.55E-03
MEBARKI_HCC_PROGENITOR_WNT_UP_BLOCKED_BY_FZD8CRD	116	0.64	1.49	1.45E-04	2.32E-03	1.78E-03

GSEA, Gene Set Enrichment Analysis; TCGA, The Cancer Genome Atlas; CESC, Cervical Cancer.

### 3.8 GO and pathway (KEGG) enrichment analysis

This study examined the relationship between 677 subtype differentially expressed genes (SSDEGs) and cervical cancer (CESC) through GO/KEGG enrichment analysis. **Table 4** shows the GO/KEGG enrichment analysis results for 78 differentially expressed subtype genes (SSDEGs).

**Table 4:** Results of GOKEGG.

ONTOLOGY	ID	Description	GeneRatio	BgRatio	pvalue	p.adjust	qvalue
BP	GO:0010951	negative regulation of endopeptidase activity	4/29	128/18870	4.21E-05	1.30E-02	9.14E-03
BP	GO:0010466	negative regulation of peptidase activity	4/29	137/18870	5.48E-05	1.30E-02	9.14E-03
BP	GO:0050921	positive regulation of chemotaxis	4/29	145/18870	6.84E-05	1.30E-02	9.14E-03
BP	GO:0052548	regulation of endopeptidase activity	5/29	288/18870	7.03E-05	1.30E-02	9.14E-03
BP	GO:0052547	regulation of peptidase activity	5/29	312/18870	1.03E-04	1.43E-02	1.01E-02
CC	GO:0034774	secretory granule lumen	4/30	322/19886	1.33E-03	4.16E-02	3.31E-02
CC	GO:0060205	cytoplasmic vesicle lumen	4/30	325/19886	1.37E-03	4.16E-02	3.31E-02
CC	GO:0031983	vesicle lumen	4/30	326/19886	1.39E-03	4.16E-02	3.31E-02
MF	GO:0002020	protease binding	4/30	142/18496	7.81E-05	5.85E-03	3.97E-03
MF	GO:0004866	endopeptidase inhibitor activity	4/30	168/18496	1.50E-04	5.85E-03	3.97E-03
MF	GO:0030414	peptidase inhibitor activity	4/30	175/18496	1.75E-04	5.85E-03	3.97E-03
MF	GO:0061135	endopeptidase regulator activity	4/30	184/18496	2.12E-04	5.85E-03	3.97E-03
MF	GO:0008237	metallopeptidase activity	4/30	185/18496	2.17E-04	5.85E-03	3.97E-03
KEGG	hsa05146	Amoebiasis	4/12	103/8876	7.88E-06	4.34E-04	2.99E-04
KEGG	hsa05219	Bladder cancer	3/12	41/8876	1.96E-05	5.38E-04	3.71E-04
KEGG	hsa05120	Epithelial cell signaling in Helicobacter pylori infection	3/12	71/8876	1.02E-04	1.88E-03	1.29E-03
KEGG	hsa04657	IL-17 signaling pathway	3/12	95/8876	2.44E-04	2.68E-03	1.85E-03
KEGG	hsa05323	Rheumatoid arthritis	3/12	95/8876	2.44E-04	2.68E-03	1.85E-03

GO, Gene Ontology; BP, Biological Process; CC, Cellular Component.

**Figure 8:**
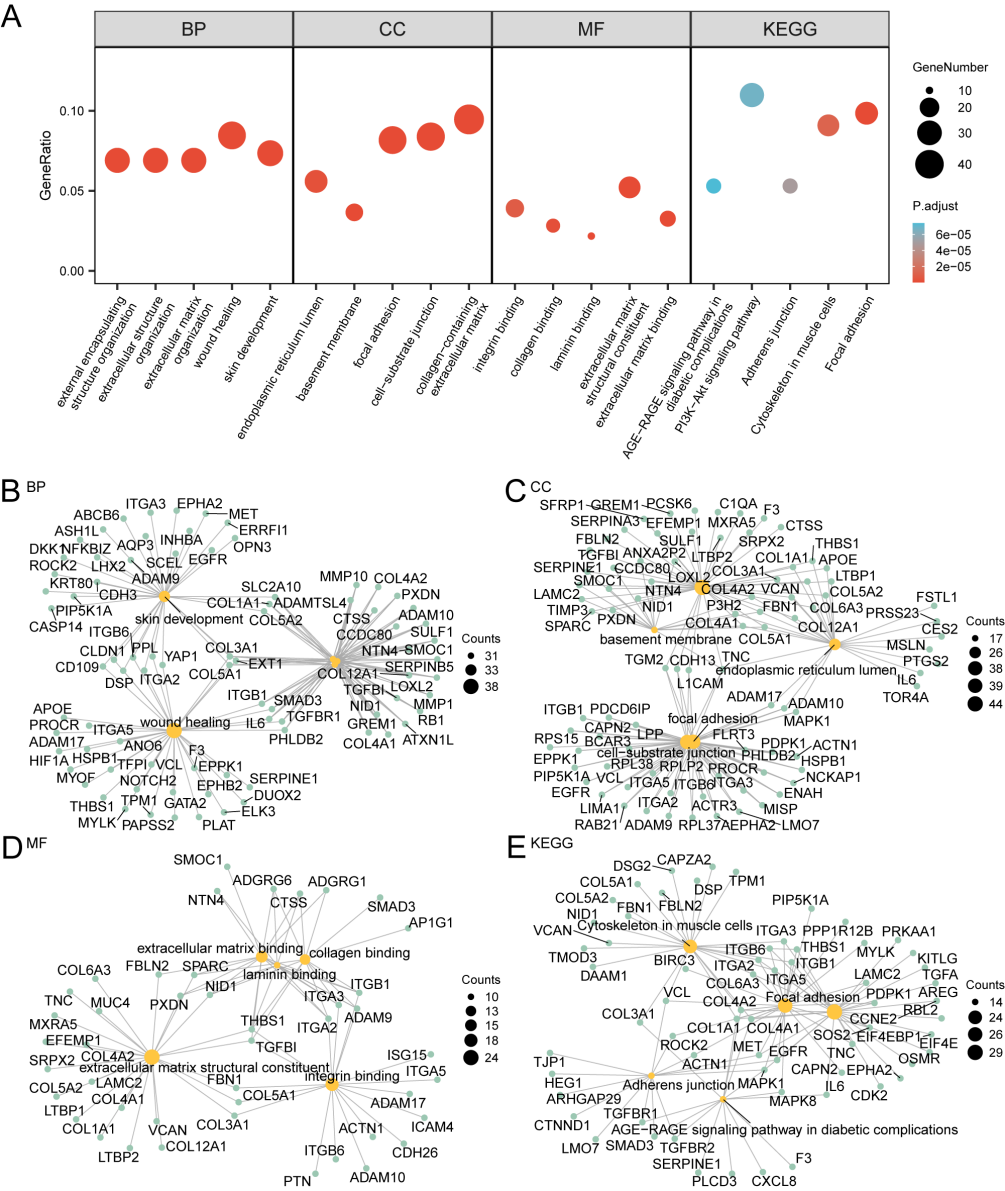
GO and KEGG enrichment analysis. **(A)** Bubble plot of GO and KEGG enrichment analysis results of subtyped differential genes (SSDEGs): BP, CC, MF and KEGG). GO terms and KEGG terms are shown on the abscissa. B-E. GO and KEGG enrichment analysis results of subtype differential genes (SSDEGs) network diagram showing BP **(B)**, CC **(C)**, MF **(D)** and KEGG **(E)**. The orange nodes represent items, the green nodes represent molecules, and the lines represent the relationship between items and molecules. The bubble size in the bubble plot represents the number of genes, and the color of the bubble represents the size of the adj.p, the redder the color, the smaller the adj. P-value, and the bluer the color, the larger the adj. P-value. The screening criteria for GO and KEGG enrichment analysis were adj.p < 0.05 and FDR value (q value) < 0.25, and the p value correction method was Benjamini-Hochberg (BH).

The results indicate that these 78 genes are mainly enriched in various biological processes, such as negative regulation of endopeptidase activity; cellular components, such as secretory granule lumen; and molecular functions, such as protease binding. They also show enrichment in multiple biological pathways. The analysis results are visualized through a bubble chart (**Figure 8A**) and a network diagram (**Figures 8B–E**), showcasing the annotations of related molecules and the number of molecules in each entry.

### 3.9 Construction of prognostic risk model for cervical cancer

**Figure 9:**
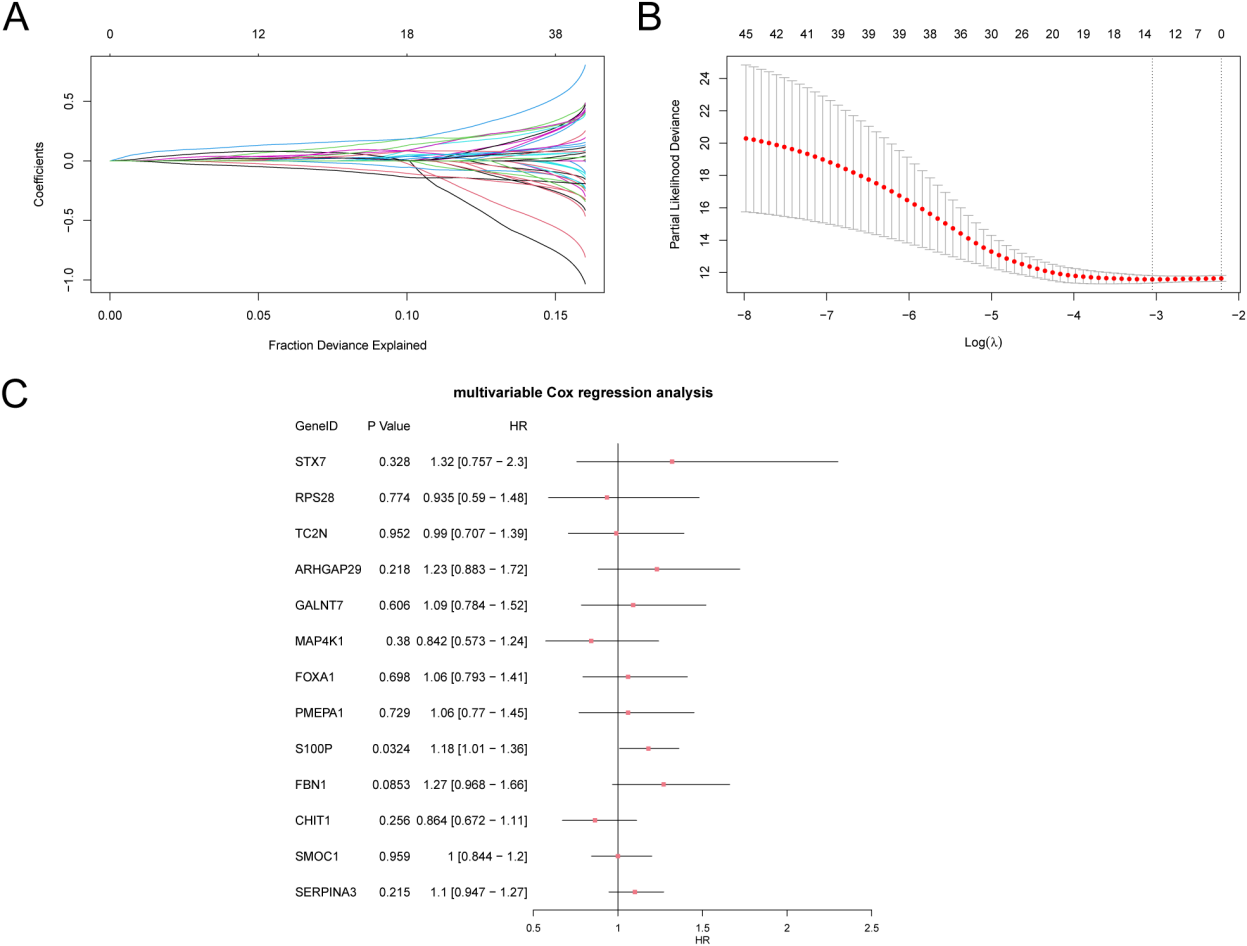
Cox regression analysis. **(A, B)**. Plots of variable trajectories of the LASSO regression model **(A)** and prognostic model **(B)**. **(C)** Forest Plot of subtype A (Cluster1) and subtype B (Cluster2) of CESC samples in the multivariate Cox regression model of 13 LASSO regression model genes in the GTEx-TCGA-CESC. LASSO (Least Absolute Shrinkage and Selection Operator).


The findings revealed that 45 SSDEGs exhibited statistical significance (p-values < 0.05) within the univariate Cox regression framework, as presented in **Supplementary Table S4**. We subsequently conducted LASSO regression analysis and constructed a LASSO regression model. We generated a trajectory plot for the LASSO variables (**Figure 9A**) alongside the diagram illustrating the LASSO regression model (**Figure 9B**) for enhanced visualization. The LASSO regression model culminated in the identification of 13 pertinent genes: *STX7, RPS28, TC2N, ARHGAP29, GALNT7, MAP4K1, FOXA1, PMEPA1, S100P, FBN1, CHIT1, SMOC1*, and *SERPINA3*. The calculation of the RiskScore was performed using the equation provided:

$$\begin{array}{l} \text{RiskScore}=STX7\;*\;\left(0.106\right)+RPS28\;*\;\left(-0.013\right)+TC2N\;*\;\left(0.0285\right)+ARHGAP29\;*\;\\ \left(0.156\right)+GALNT7\;*\;\left(0.055\right)+MAP4K1\;*\;\left(-0.074\right)+FOXA1\;*\;\left(0.019\right)+PMEPA1\;*\;\\ \left(0.072\right)+S100P\;*\;\left(0.046\right)+FBN1\;*\;\left(0.066\right)+CHIT1\;*\;\left(-0.051\right)+SMOC1\;*\;\left(0.024\right)+\\ SERPINA3\;*\;\left(0.032\right). \end{array}$$

Finally, we performed multivariate Cox analysis on the genes from the LASSO regression model and created a forest plot (**Figure 9C**).

### 3.10 Prognostic analysis of cervical cancer prognostic risk model

**Figure 10:**
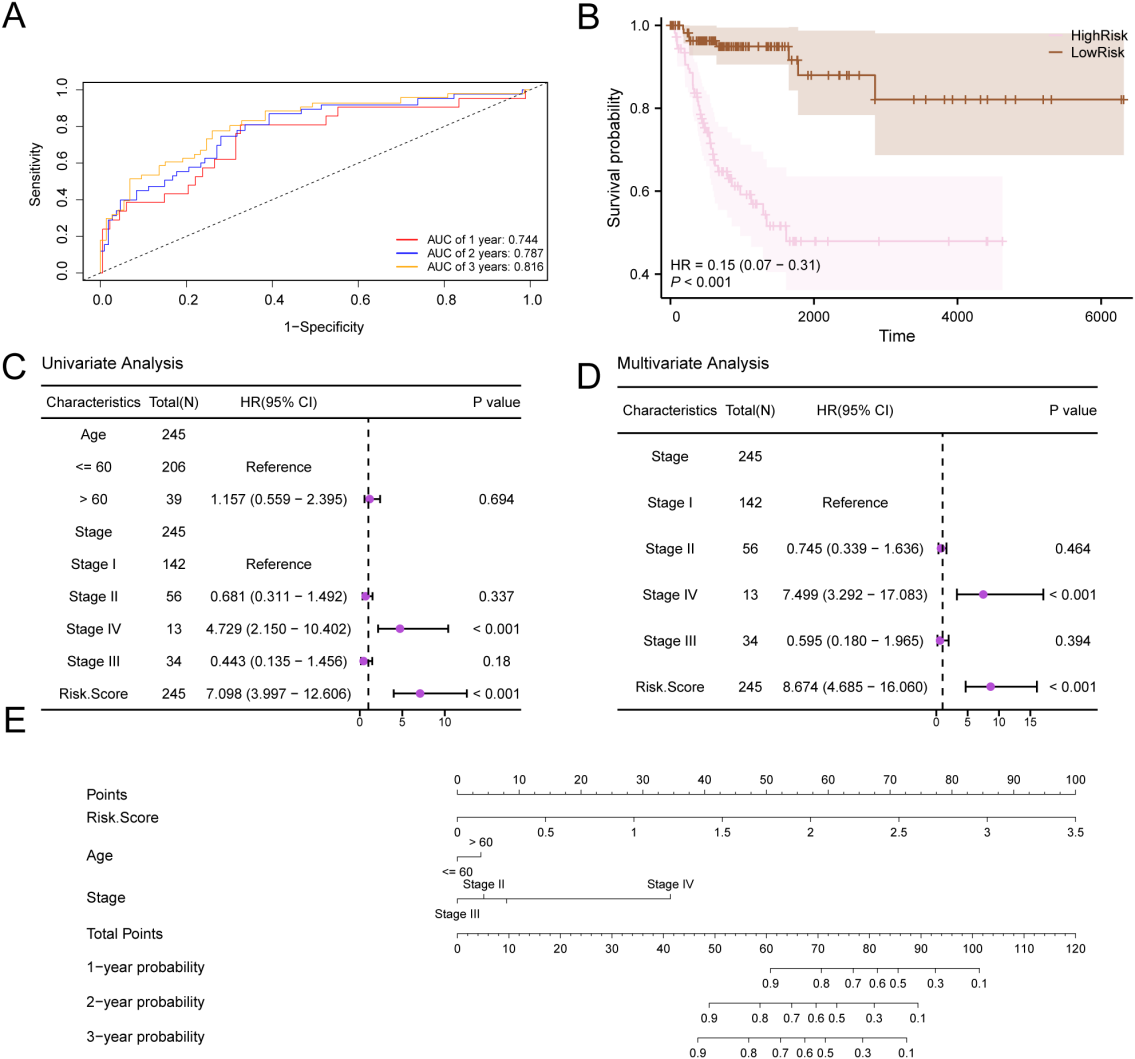
Prognostic analysis. **(A)** Time-dependent ROC curves of the CESC group in the GTEx-TCGA-CESC. **(B)** Prognostic KM curve between RiskScore high and low groups and d DFS of the CESC sample. **(C, D)** Forest Plot of RiskScore and clinical information in univariate Cox regression model **(C)** and multivariate Cox regression model **(D)**. **(E)** Nomogram of RiskScore and clinical information in univariate and multivariate Cox regression model. p value < 0.001, highly statistically significant.

First, we created ROC curves that vary over time for the CESC samples in the GTEx-TCGA-CESC dataset (**Figure 10A**). These curves indicated a moderate accuracy for the prognostic risk model, with AUC values between 0.7 and 0.9 for 1, 2, and 3 years. Next, we conducted a Kaplan-Meier curve analysis for DFS in the CESC samples from the GTEx-TCGA-CESC dataset (**Figure 10B**). This analysis revealed a highly significant difference in DFS between the High-Risk and Low-Risk groups, with a p-value of less than 0.001.The forest plots (**Figures 10C, D**) illustrate the univariate and multivariate Cox regression analysis results, while detailed findings are presented in **Table 5**. These plots highlight the significance of RiskScore and clinical staging, both of which have p-values less than 0.01. We constructed a nomogram (**Figure 10E**) that illustrated the contributions of RiskScore, age, and clinical staging to the prognostic risk model.

**Table 5:** Results of Cox analysis.

Characteristics	Total (N)	HR (95% CI) Univariate analysis	P value Univariate analysis	HR (95% CI) Multivariate analysis	P value Multivariate analysis
Age	245				
<= 60	206	Reference			
> 60	39	1.157 (0.559-2.395)	0.694		
Stage	245				
Stage I	142	Reference		Reference	
Stage II	56	0.681 (0.311-1.492)	0.337	0.745 (0.339-1.636)	0.464
Stage IV	13	4.729 (2.150-10.402)	< 0.001	7.499 (3.292-17.083)	< 0.001
Stage III	34	0.443 (0.135-1.456)	0.180	0.595 (0.180-1.965)	0.394
Risk.Score	245	7.098 (3.997-12.606)	< 0.001	8.674 (4.685-16.060)	< 0.001

HR > 1 indicates that the variable is a risk factor, and HR < 1 is a protective factor. Univariate p values < 0.1 were included in the analysis.

**Figure 11:**
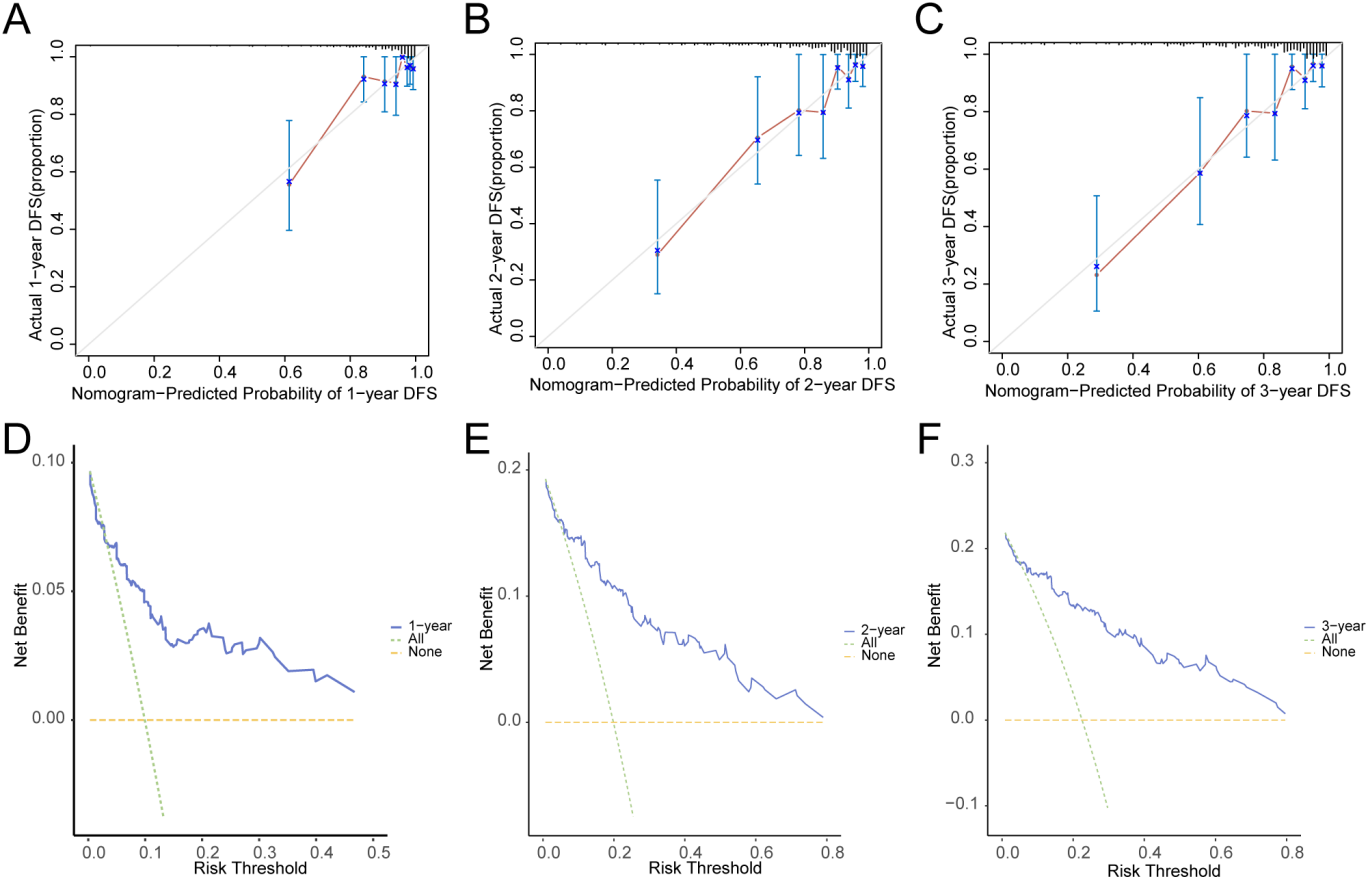
Prognostic analysis. **(A–C)**. Calibration curve of 1 year **(A)**, 2 years **(B)**, and 3 years **(C)** of the prognostic risk model for CESC. D-F. 1-year **(D)**, 2-year **(E)**, and 3-year **(F)** decision curve analysis (DCA) plots of the prognostic risk model for CESC.

In addition, we conducted a prognostic calibration analysis of the prognostic risk model for cervical cancer (CESC) for 1 year, 2 years, and 3 years, and plotted the calibration curves (**Figures 11A–C**). The results show that the prognostic risk model for cervical cancer (CESC) has the best clinical predictive effect for 3 years. Finally, we evaluated the clinical utility of the prognostic risk model for cervical cancer (CESC) at 1, 2, and 3 years through decision curve DCA and presented the results (**Figures 11D–F**).

### 3.11 Model validation for dataset GSE44001

**Figure 12:**
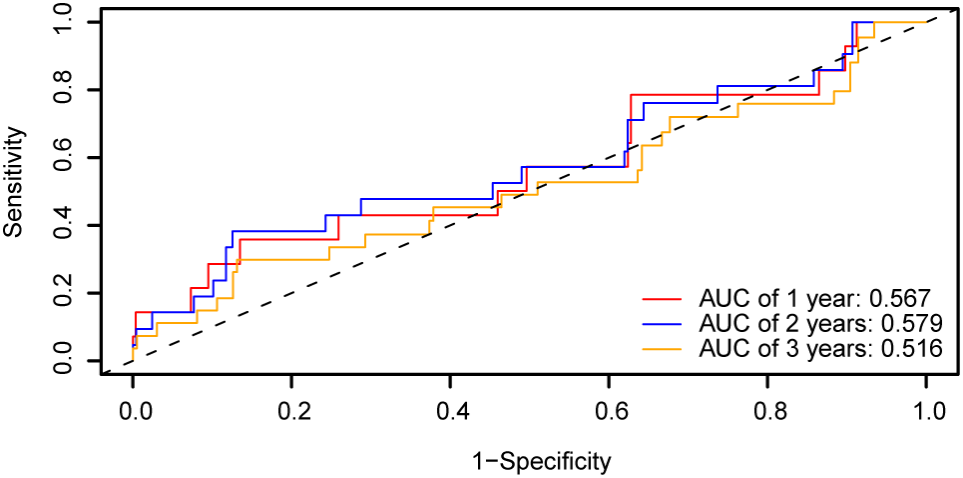
Prognostic analysis. Time-dependent ROC curve of the CESC group in dataset GSE44001. ROC, Receiver Operating Characteristic Curve; AUC, Area Under the Curve.

Based on the risk coefficient calculation of the dataset GSE44001 using LASSO regression analysis. Subsequently, we plotted the time-dependent ROC curve (**Figure 12**) for the cervical cancer group (CESC) in the cervical cancer dataset. The results indicate that the prognostic risk model for cervical cancer (CESC) presents AUC values between 0.5 and 0.7 across 1, 2, and 3 years.

### 3.12 GSVA

**Figure 13:**
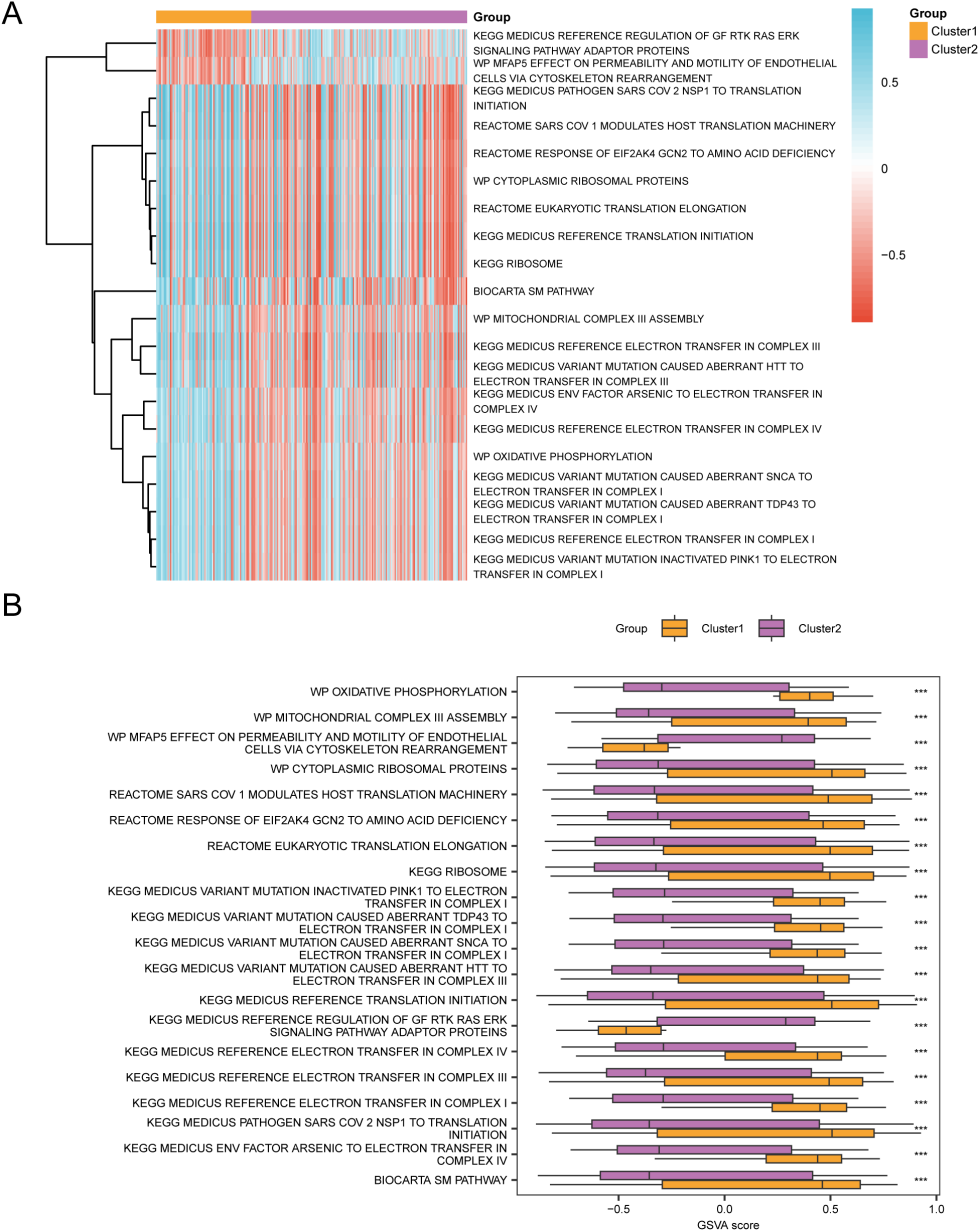
GSVA analysis. **(A, B)**. Heat map **(A)** and group comparison map **(B)** of gene set variation analysis (GSVA) results between subtype B (Cluster2) group and subtype A (Cluster1) group of cervical cancer (CESC) samples in the TCGA-CESC. GSVA, Gene Set Variation Analysis. *** represents p value < 0.001, highly statistically significant. Purple represents subgroup B (Cluster2) and orange represents subgroup A (Cluster1). The screening criteria for gene set variation analysis (GSVA) was adj.p < 0.05, and the p value correction method was Benjamini-Hochberg (BH). In the heat map, blue represents low enrichment and red represents high enrichment.

Gene set variation analysis (GSVA) was conducted on all genes from cervical cancer (CESC) samples in the GTEx-TCGA-CESC dataset. This analysis aimed to identify differences in the c2.cp.v2023.2.Hs.symbols.gmt gene set between subtype B (Cluster 2) and subtype A (Cluster 1).Detailed information regarding the gene set analysis is presented in **Table 6**. Next, the top 20 pathways were selected based on an adj.p < 0.05. These pathways were sorted in descending order by the absolute value of logFC. The differential expression of these pathways between subtype B (Cluster 2) and subtype A (Cluster 1) was then visualized in **Figure 13A**.

**Table 6:** Results of GSVA.

Pathway	logFC	AveExpr	t	P.Value	adj.P.Val	B
KEGG MEDICUS REFERENCE ELECTRON TRANSFER IN COMPLEX I	-0.56051	-0.00539	-6.88	4.34E-11	2.20E-08	14.82
KEGG MEDICUS VARIANT MUTATION INACTIVATED PINK1 TO ELECTRON TRANSFER IN COMPLEX I	-0.56009	-0.0041	-6.94	2.90E-11	2.20E-08	15.21
KEGG MEDICUS ENV FACTOR ARSENIC TO ELECTRON TRANSFER IN COMPLEX IV	-0.55983	-0.02649	-7.06	1.42E-11	1.52E-08	15.89
KEGG MEDICUS REFERENCE TRANSLATION INITIATION	-0.55751	-0.00503	-5.36	1.76E-07	5.41E-06	6.91
KEGG MEDICUS REFERENCE ELECTRON TRANSFER IN COMPLEX III	-0.55563	-0.02906	-5.86	1.38E-08	8.60E-07	9.32
KEGG MEDICUS VARIANT MUTATION CAUSED ABERRANT TDP43 TO ELECTRON TRANSFER IN COMPLEX I	-0.55099	-0.00589	-6.84	5.48E-11	2.38E-08	14.60
WP MITOCHONDRIAL COMPLEX III ASSEMBLY	-0.54996	-0.03923	-6.59	2.34E-10	4.62E-08	13.21
KEGG MEDICUS PATHOGEN SARS COV 2 NSP1 TO TRANSLATION INITIATION	-0.54622	-0.00867	-5.24	3.23E-07	8.34E-06	6.33
KEGG RIBOSOME	-0.54398	-0.00693	-5.42	1.31E-07	4.49E-06	7.18
BIOCARTA SM PATHWAY	-0.53856	-0.01842	-5.47	1.01E-07	3.62E-06	7.43
REACTOME EUKARYOTIC TRANSLATION ELONGATION	-0.53642	-0.00788	-5.37	1.69E-07	5.29E-06	6.94
KEGG MEDICUS REFERENCE ELECTRON TRANSFER IN COMPLEX IV	-0.53455	-0.02004	-6.49	4.11E-10	6.72E-08	12.67
KEGG MEDICUS VARIANT MUTATION CAUSED ABERRANT SNCA TO ELECTRON TRANSFER IN COMPLEX I	-0.53331	0.000449	-6.61	2.09E-10	4.54E-08	13.32
KEGG MEDICUS VARIANT MUTATION CAUSED ABERRANT HTT TO ELECTRON TRANSFER IN COMPLEX III	-0.52911	-0.01911	-5.87	1.31E-08	8.28E-07	9.38
KEGG MEDICUS REFERENCE REGULATION OF GF RTK RAS ERK SIGNALING PATHWAY ADAPTOR PROTEINS	0.526745	-0.02655	6.88	4.32E-11	2.20E-08	14.83
REACTOME RESPONSE OF EIF2AK4 GCN2 TO AMINO ACID DEFICIENCY	-0.52632	-0.01099	-5.58	6.00E-08	2.53E-06	7.93
WP CYTOPLASMIC RIBOSOMAL PROTEINS	-0.52282	-0.00643	-5.32	2.23E-07	6.48E-06	6.68
REACTOME SARS COV 1 MODULATES HOST TRANSLATION MACHINERY	-0.52162	-0.01123	-5.09	6.73E-07	1.54E-05	5.64
WP OXIDATIVE PHOSPHORYLATION	-0.51567	-0.00306	-6.78	7.58E-11	2.71E-08	14.29
WP MFAP5 EFFECT ON PERMEABILITY AND MOTILITY OF ENDOTHELIAL CELLS VIA CYTOSKELETON REARRANGEMENT	0.515332	-0.03125	7.20	6.29E-12	1.52E-08	16.67

GSVA, Gene Set Variation Analysis.

The disparities were then validated using the Mann-Whitney U test, and the comparative results were illustrated in the diagram presented in **Figure 13B**. The gene set variation analysis (GSVA) revealed that the MEDICUS VARIANT MUTATION INACTIVATED PINK1 TO ELECTRON TRANSFER IN COMPLEX I, MEDICUS REFERENCE ELECTRON TRANSFER IN COMPLEX I, MEDICUS VARIANT MUTATION CAUSED ABERRANT TDP43 TO ELECTRON TRANSFER IN COMPLEX I, MEDICUS VARIANT MUTATION CAUSED ABERRANT SNCA TO ELECTRON TRANSFER IN COMPLEX I, OXIDATIVE PHOSPHORYLATION, MEDICUS REFERENCE ELECTRON TRANSFER IN COMPLEX IV, MEDICUS ENV FACTOR ARSENIC TO ELECTRON TRANSFER IN COMPLEX IV, MEDICUS VARIANT MUTATION CAUSED ABERRANT HTT TO ELECTRON TRANSFER IN COMPLEX III, MEDICUS REFERENCE ELECTRON TRANSFER IN COMPLEX III, MITOCHONDRIAL COMPLEX III ASSEMBLY, BIOCARTA SM PATHWAY, RIBOSOME, MEDICUS REFERENCE TRANSLATION INITIATION, EUKARYOTIC TRANSLATION ELONGATION, CYTOPLASMIC RIBOSOMAL PROTEINS, RESPONSE OF EIF2AK4 GCN2 TO AMINO ACID DEFICIENCY, SARS COV 1 MODULATES HOST TRANSLATION MACHINERY, MEDICUS PATHOGEN SARS COV 2 NSP1 TO TRANSLATION INITIATION, and MFAP5 EFFECT ON PERMEABILITY AND MOTILITY OF ENDOTHELIAL CELLS VIA CYTOSKELETON REARRANGEMENT, MEDICUS REFERENCE REGULATION OF GF RTK RAS ERK SIGNALING PATHWAY ADAPTOR PROTEINS in the subtype B (Cluster 2) and subtype A (Cluster 1) groups were statistically significant.

## 4 Discussion

In recent years, considerable attention has been directed towards the rates of occurrence and mortality associated with CESC.A report from the American Cancer Society, the annual incidence of cervical cancer among women aged 30 to 44 in the United States increased by 1.7% from 2012 to 2019 (32). Despite advancements in cervical cancer treatment (33), numerous factors continue to influence the options available, such as tumor size, invasion depth, and lymphovascular invasion. Consequently, the exploration of new biological targets is critical for driving breakthroughs in healthcare.

This study amalgamated diverse omics data related to cervical cancer, identifying seven pivotal genes: *PCBP3, ARNT, ANP32E, DSTN, CD2AP, EPAS1*, and *ACTN1*. Moreover, consensus clustering analysis unveiled two distinct subtypes of cervical cancer, designated as Cluster 1 and Cluster 2, which displayed remarkable differences in disease-free survival. The prognostic risk model established through LASSO regression analysis identified 13 genes—*STX7, RPS28, TC2N, ARHGAP29, GALNT7, MAP4K1, FOXA1, PMEPA1, S100P, FBN1, CHIT1, SMOC1*, and *SERPINA3*—that exhibited strong predictive capabilities at multiple time intervals. These discoveries furnish novel insights into personalized treatment strategies for cervical cancer and establish a foundation for further investigation into the possible involvement of disulfide-dependent mechanisms of cell death in cancer therapy.

The identification of DEGs in cervical squamous cell carcinoma (CESC) elucidates the underlying mechanisms of the tumor. Among the seven key genes we identified, *PCBP3* is notable for its role in regulating both cell proliferation and apoptosis, potentially promoting tumor growth by stabilizing mRNA that encourages cell division, making it a promising target for therapy (34). Another important gene, *ARNT*, is integral to the hypoxia-inducible factor (HIF) signaling pathway, which allows cells to adapt to low-oxygen conditions commonly found in solid tumors (35, 36). The increased expression of *ARNT* in cervical cancer suggests that the tumor is responding to its microenvironment, facilitating processes like angiogenesis and metabolic changes that support tumor survival and growth. Additionally, *ANP32E* has been implicated in breast cancer, where it inhibits cell proliferation and encourages apoptosis; thus, its downregulation may lead to more aggressive tumor behavior (37–39). The differences in *ANP32E* expression in esophageal cancer, when compared to other cancers, may indicate a potential therapeutic target (40), offering critical insights for the formulation of treatment strategies in cervical cancer.

Consensus clustering analysis has identified two subtypes of cervical cancer, which significantly enhances our understanding of the disease’s heterogeneity. Each subtype exhibits unique clinical characteristics, underscoring the necessity for treatments tailored to each specific subtype. Kaplan-Meier analysis has shown significant differences in disease-free survival rates, indicating that these molecular subtypes are associated with varying clinical outcomes (41–43). Additionally, variations in the expression of pivotal genes such as *PCBP3, ACTN1, and EPAS1* highlight the biological diversity within these subtypes. In relation to the immune microenvironment, CIBERSORT analysis has uncovered differences in immune cell infiltration. Specifically, a negative correlation exists between certain genes and immune cells, implying that these genes may influence the regulation of immune responses (44–46). Exploring the immune characteristics of these cervical cancer subtypes could be instrumental in developing effective immunotherapy strategies.

The prognostic risk model developed using 13 genes identified through LASSO regression demonstrates significant potential for clinical application, effectively stratifying patients based on their risk characteristics. To assess the model’s ability to predict patient prognosis, we used the AUC as a metric, where a higher AUC value signifies better classification performance and greater comparability of features. The prognostic model for bladder cancer developed by Deng et al. is associated with disulfide-induced apoptosis. The prognostic model for bladder cancer developed by Deng et al. is associated with disulfidptosis, and they reported higher AUC values (47). Similarly, the renal cell carcinoma model involving disulfidptosis also reported high AUC values (48).

We also conducted calibration analyses of the risk assessment prognostic assessment model for cervical cancer (CESC) over periods of 1 to 3 years, with results indicating that the model’s predictions were most accurate at the 3-year mark. We evaluated the clinical utility of the CESC model using Decision curve analysis to assess its performance over one, two, and three years. Here, we present the results. Our findings show a decline in effectiveness over three, two, and one-year periods, confirming that the prognostic model based on DRGs is reliable and accurate. This indicates that the prognostic model based on Diagnosis-Related Groups (DRGs) is both reliable and accurate.

When the model’s line remains above both “All Positive” and “All Negative” for a wider range, it indicates greater net benefits and improved performance. The LASSO regression model we developed has the highest clinical predictive performance at three years, followed by two years, and the lowest at one year. Our research indicates that DRGs have potential targeted therapeutic effects for high-risk cervical cancer patients.

GSEA analysis revealed several significantly enriched pathways, particularly the HCC Progenitor Wnt Upregulated and NF-κB Targets Keratinocyte Upregulated pathways. The Wnt signaling pathway is integral to cellular proliferation and differentiation; however, its aberrant activation can result in tumorigenesis and metastasis (49–51). Consequently, inhibiting Wnt signaling presents potential new therapeutic strategies. Additionally, the NF-κB pathway is crucial for regulating inflammatory responses and cell survival, contributing to tumor progression and the resistance of cancer cells to apoptosis (52). Targeting these pathways may not only inhibit tumor growth but also increase the sensitivity of cancer cells to existing treatments. Nevertheless, the interplay between these signaling pathways, along with their interconnections with other pathways, necessitates further exploration. For instance, the activation of the NF-κB pathway may augment Wnt signaling, potentially creating a feedback loop that fosters tumorigenesis (53). This observation implies that multi-targeted approaches could be more efficacious than single-target strategies, highlighting the intricate nature of tumor biology and the need for integrated methodologies in drug development. The modulation of pathway activity—whether through inhibition or activation—can markedly affect tumor cell behavior.

Specifically, cell migration and invasion can be reduced by suppressing Wnt signaling (54–56). A thorough understanding of these dynamics is essential for devising effective treatment strategies that can navigate resistance mechanisms and improve patient outcomes.

This study’s analysis of immune infiltration underscores the importance of the interactions among various immune cells, which could have significant implications for treatment strategies in cervical cancer. Immunoinfiltration analysis showed a significant negative correlation between *CD8 T* cells and resting memory *CD4 T* cells in different groups (r = -0.463). *CD8 T* cells can act as key effector cells responsible for targeting and eliminating tumor cells. Resting memory *CD4 T* cells play a key role in regulating immune responses and maintaining immune memory (57, 58). There is competition between *CD8 T* cells and resting memory *CD4 T* cells, which may affect the efficacy of immunotherapy. Additionally, the negative correlation between *DSTN* and *CD8 T* cells (r = -0.285) indicates that *DSTN* may regulate the infiltration process of immune cells. Since *DSTN* is related to cytoskeletal dynamics, it may influence the movement and efficacy of CD8 T cells in the tumor microenvironment. Understanding the relationship between essential genes and immune cell infiltration may help identify biomarkers for personalized immunotherapy (59). This research underscores the urgent need to explore the interactions among immune cells and their effects on anti-tumor responses to refine existing immunotherapies and enhance their effectiveness in treating cervical cancer. Future research should focus on exploring combination strategies that increase *CD8 T* cells while reducing resting memory *CD4 T* cells, ultimately improving patient prognosis (60, 61).

Our model requires further experimental validation to assess its clinical applicability. To create a stronger predictive model, the role of DRGs in cervical cancer prognosis and their correlation with clinical features must be experimentally validated. Although our data provide valuable insights, it is crucial to obtain additional experimental evidence to substantiate these findings and enhance the application of these two genes in targeted therapy for cervical cancer. Furthermore, relying on a single dataset for analysis may introduce biases, as it might not accurately represent a wider population or account for variations across different contexts. Such limitations highlight the imperative for additional research to corroborate the results and augment their clinical significance.

## 5 Conclusion

We identified seven key genes and constructed two distinct subtypes of cervical cancer. By utilizing thirteen genes, we developed a prognostic risk model that demonstrates strong predictive capabilities over time. This model holds significant promise for clinical applications. Future work should focus on expanding the sample size and conducting cross-platform validation to ensure that the conclusions are robust and reliable.

## Data Availability

The datasets presented in this study can be found in online repositories. The names of the repository/repositories and accession number(s) can be found in the article/**Supplementary Material**.
